# Meta-Analysis of Bovine Digital Dermatitis Microbiota Reveals Distinct Microbial Community Structures Associated With Lesions

**DOI:** 10.3389/fcimb.2021.685861

**Published:** 2021-07-16

**Authors:** Ben Caddey, Jeroen De Buck

**Affiliations:** Department of Production Animal Health, Faculty of Veterinary Medicine, University of Calgary, Calgary, AB, Canada

**Keywords:** cattle, digital dermatitis, microbiota, skin microbiome, *Treponema*, meta-analysis

## Abstract

Bovine digital dermatitis (DD) is a significant cause of infectious lameness and economic losses in cattle production across the world. There is a lack of a consensus across different 16S metagenomic studies on DD-associated bacteria that may be potential pathogens of the disease. The goal of this meta-analysis was to identify a consistent group of DD-associated bacteria in individual DD lesions across studies, regardless of experimental design choices including sample collection and preparation, hypervariable region sequenced, and sequencing platform. A total of 6 studies were included in this meta-analysis. Raw sequences and metadata were identified on the NCBI sequence read archive and European nucleotide archive. Bacterial community structures were investigated between normal skin and DD skin samples. Random forest models were generated to classify DD status based on microbial composition, and to identify taxa that best differentiate DD status. Among all samples, members of *Treponema, Mycoplasma, Porphyromonas*, and *Fusobacterium* were consistently identified in the majority of DD lesions, and were the best genera at differentiating DD lesions from normal skin. Individual study and 16S hypervariable region sequenced had significant influence on final DD lesion microbial composition *(P* < 0.05). These findings indicate that members of *Treponema, Mycoplasma, Porphyromonas*, and/or *Fusobacterium* may have significant roles in DD pathogenesis, and should be studied further in respect to elucidating DD etiopathogenic mechanisms and developing more effective treatment and mitigation strategies.

## Introduction

Bovine digital dermatitis (DD) is an infectious skin lesion that was first reported in Italy in 1974 (Cheli and Mortellaro, 1974), and is a significant cause of lameness in cattle across the world (Read and Walker, 1998). DD is a multifaceted disease, having both painful ulcerative lesions and chronic hyperkeratotic stages across the different morphological presentations of the lesions (Döpfer et al., 1997; Berry et al., 2012). Treatment and production costs associated with DD lead to significant losses for both beef and dairy producers mainly due to either decreased average daily gain and decreased milk production (Gomez et al., 2015; Davis-Unger et al., 2017; Kulow et al., 2017; Dolecheck et al., 2019). Prevalence of DD at the herd level is reported to be as high as 93% (Solano et al., 2016), but prevalence estimates vary based on a multitude of factors including parity, breed, housing type and hoof trimming frequency (Holzhauer et al., 2006; Solano et al., 2016; Bran et al., 2018; Yang et al., 2019). Current treatment and prevention strategies rely on topical applications and footbaths of broad spectrum antimicrobial agents, most commonly tetracycline or copper sulfate, and are largely ineffective at returning lesions to normal skin and preventing recurrence of active lesions (Krull et al., 2016; Jacobs et al., 2018).

Early attempts to determine etiological agents of DD pointed to *Treponema* as the main pathogen of these lesions. More recent evidence shifts the focus to studying the roles of both *Treponema* and additional anaerobic bacteria in lesion formation and progression, leading to a polymicrobial hypothesis of DD causation (Yano et al., 2010; Santos et al., 2012). The quantities of *Treponema* are dramatically higher in most DD lesions compared to healthy skin; however, there exists a wide variety of *Treponema* species and phylotypes across individual animals and between studies (Klitgaard et al., 2008; Klitgaard et al., 2013; Krull et al., 2014). Most commonly, *Treponema phagedenis, T. medium, T. refringens, T. denticola*, and *T. pedis* are identified in DD lesions, but presence and population dynamics of each species varies across studies (Krull et al., 2014; Zinicola et al., 2015a; Zinicola et al., 2015b).

Additional bacteria outside the genus *Treponema* likely play some role in DD pathogenesis based on their presence and abundance within DD lesions; however, the conclusions on which of these bacteria potentially are involved can vary dramatically across studies of DD microbiota. In addition to *Treponema*, many recent metagenomic studies associate *Mycoplasma* abundance with DD lesions (Krull et al., 2014; Nielsen et al., 2016; Hesseling et al., 2019). Inconsistencies most notably arise between studies when implicating other bacterial genera in DD pathogenesis. *Porphyromonas* (Zinicola et al., 2015b; Nielsen et al., 2016)*, Fusobacterium* (Nielsen et al., 2016; Moreira et al., 2018), and *Dichelobacter* (Krull et al., 2014; Moreira et al., 2018), among a few other genera are inconsistently associated with DD lesion microbiota and pathogenesis. It is currently unknown if these major differences between metagenomic studies are due to differences in sequencing, bioinformatic processing, sample size or if DD lesion microbiota differs across beef and dairy cattle breeds.

Comparing conclusions between metagenomic studies can lead to erroneous interpretations, as variation in study objectives and analytical processing can influence findings and result in contradictory comparisons between study outcomes. Many metagenomic studies process individual sequencing reads into operational taxonomic units, which are not directly comparable across experiments and can be a source of variation when drawing comparisons between studies (Callahan et al., 2017). Primer bias in deep amplicon sequencing can also have a significant impact on observed bacterial populations, as certain primers used in the amplification process before sequencing might not amplify all bacteria at equal rates, but the extent of this impact is not determined yet in DD-specific microbial communities. In addition, different study designs, experimental conditions, and statistical analyses can significantly alter the results and conclusions on microbial composition (Goodrich et al., 2014). Therefore, a meta-analysis is required in order to draw controlled and quantitative conclusions across these metagenomic DD studies.

The differences in findings between DD metagenomic studies leads to a lack of consensus on which bacteria may be potential pathogens, which is a limiting factor for future DD pathogenesis research. The goal of this meta-analysis was to use a consistent analytical approach on pooled sequences from individual DD metagenomic studies to identify a DD-associated microbiota that consistently and accurately differentiates DD lesions from normal skin. Identifying a consistent DD-associated microbiota, regardless of individual animal variation, 16S hypervariable region sequenced, cattle breed, and other study design choices would provide strong evidence for a potential core DD microbiota and initiate and accelerate future targeted research efforts toward determining etiopathogenesis mechanisms of DD.

## Materials and Methods

### Data Collection and Selection Criteria

Searches for publicly available data were performed on the NCBI Sequence Read Archive (SRA) database and the European Nucleotide Archive using the search term “digital dermatitis”, and limiting search results by “BioProject”. BioProject accession numbers (study identifiers) containing high throughput sequencing reads and associated metadata were collected. For inclusion in the meta-analysis, all studies had to have publicly available sequencing reads before September 1, 2020. Studies were required to be a bovine DD metagenomic study, and have conducted deep amplicon sequencing of a 16S rRNA hypervariable region using universal bacterial primers on samples originating from bovine skin. Raw data (DNA sequences and associated quality scores) and sufficient metadata (containing information differentiating samples by DD diagnosis) had to be publicly available for inclusion in the meta-analysis. Additional information on sample collection was obtained for each study. All samples were collected with biopsy punches from feet cleaned with water or chlorhexidine of either live animals or at a slaughterhouse. Depth and exact location of skin lesion samples were not universally available across all studies. Other relevant details concerning experimental processing of samples before sequencing are described in **Table 1**. Fastq files were all downloaded from the NCBI SRA database (www.ncbi.nlm.nih.gov/sra).

**Table 1:** Characteristics of studies and sequencing experiments included in meta-analysis.

Study	Objectives	Conclusions	16S rRNA region	DNA extraction kit	Platform	Sample size/lesion stages	# Reads^b^	Operation type/country	BioProject ID
Gotoh et al., 2020	Compare microbiota before and after treatments.	*Treponema* relative abundance is reduced upon treatment.	V1V3	Dneasy Blood and Tissue Kit	LS454	32/Active	411,834	Dairy/Japan	PRJDB5495
Nielsen et al., 2016	Determine what microbiota is positively associated with DD.	*Treponema*, *Mycoplasma*, *Fusobacterium* and *Porphyromonas are* positively associated with DD.	T-V3V4^a^ & V1V2	AllPrep DNA/RNA/miRNA Kit	Illumina	68^e^/Healthy, Inactive	2,428,737 (V1V2)	Dairy/Denmark	PRJNA300499
							1,613,962 (T-V3V4^a^)		
Hesseling et al., 2019	Determine the prevalence of DD and identify which bacteria are consistent in DD lesions.	DD prevalence is significantly higher in dairy breeds than beef cattle. *Treponema are* significantly more abundant in DD compared to normal skin.	V3V4	N/A^c^	Illumina	18^e^/Healthy, Active, Inactive	2,714,524	Mixed^d^/Australia	PRJNA429866
Beninger et al., 2018	Quantify the abundances of the four *Treponema* species in DD lesions.	*Treponema* species composition and quantities correlate with DD lesion stages.	V1V2	Dneasy Blood and Tissue Kit	Illumina	16^f^/Active	298,391^c^	Dairy/Canada	PRJNA478809
Moreira et al., 2018	Describe DD microbiota in year-round grazing dairy cattle.	DD lesion progression is associated with *Treponema* species and *Dichelobacter nodosus*.	T-V3V4 & V1V2	Dneasy Blood and Tissue Kit	Illumina	92/Healthy, DD^g^	4,958,950	Dairy/Brazil	PRJNA369034
Caddey et al., submitted for publication	Identify and describe bacterial populations associated with DD lesions.	DD lesions were associated with species of *Treponema, Mycoplasma, Porphyromonas, Fusobacterium*, and *Bacteroides*	V3V4	Dneasy Blood and Tissue Kit	Illumina	98/Healthy, Active, Inactive	4,860,255^c^	Beef/Canada	PRJNA664530

a T-V3V4 refers to primers selectively amplifying Treponema within the V3V4 hypervariable region.

b Number of reads represents the published total number of sequencing reads after quality filtering.

c Not reported in BioProject description or associated publication. The number shown originates from the total number of reads after quality filtering in the current meta-analysis.

d Skin samples came from both dairy and beef breeds. Metadata was not sufficient to link operation type to individual samples.

e Skin samples came from slaughterhouse animals instead of on farm.

f Skin samples were disinfected with Chlorhexidine prior to sampling.

g Individual lesion stages were not reported.

### Sequence Processing

Raw fastq files that contained reads from both the V1V2 and *Treponema-*specific V3V4 16S rRNA regions were processed with Bowtie2 v.2.3.5.1 (Langmead and Salzberg, 2012) in order to separate reads by hypervariable region. Afterwards, sequences were processed using the DADA2 R package v.1.14.1 (Callahan et al., 2016). Quality filtering and base call error models were conducted on each study independently, in order to account for differences in error rates between sequencing runs. Reads were truncated after the average Phred score dropped below 30, and any reads with ambiguous nucleotides were removed. The recommended DADA2 settings for each sequencing platform (Illumina and LS454) were used to infer amplicon sequence variants (ASVs). Taxonomy was assigned using the DADA2 naïve Bayesian classifier against the SILVA v.138 database (Quast et al., 2013). Prior to analysis, all ASVs classified as Eukaryota, Mitochondria, or Chloroplast were removed.

### Data Analysis

Due to the lack of consistent information across studies on individual lesion stages, all samples included in analysis were categorized as “DD positive” or “DD negative”. DD positive samples were defined as any sample from DD lesion skin at any stage of the disease. DD negative samples were classified as any sample from skin with no visible DD lesion present. Any samples that were obtained from treated lesions were removed from downstream analysis. For diversity analyses, samples with less than 1500 reads were removed, and the remaining samples were rarefied down to the minimum sequencing depth. Observed ASV count, Chwfi 2ao1 richness estimate, Fisher’s alpha and Shannon’s diversity index were used to evaluate diversity (richness and evenness) within DD positive and negative samples. Analysis of variance (ANOVA) followed by *post hoc* Tukey’s Honest Significant Difference (TukeyHSD) test determined any significant differences of diversity between DD statuses. For beta diversity analyses, ASVs were grouped at phylum, family, and genus taxonomic levels, and Bray-Curtis dissimilarities between samples were explored using Principal Coordinates (PCoA) analysis. Permutational ANOVA (PERMANOVA) with 999 permutations, followed by a test for multivariate homogeneity of group dispersions (BETADISP) was performed to identify differences in microbial composition between DD negative and DD positive samples.

The influence of different study variables and experimental designs on microbial composition was measured exclusively on DD positive samples by repeating the diversity analysis methods described above. Alpha diversity and relative abundance plots evaluated the effect of different 16S rRNA hypervariable regions sequenced, and beta diversity measured the variation in microbial composition by individual study, 16S rRNA hypervariable region, and sequencing platform. All diversity metrics and statistical analyses were conducted using the vegan package (v. 2.5.6) in R (Dixon, 2003).

Random forest modeling was used to predict DD status based on microbial composition, and identify the microbiota that best differentiates DD positive from DD negative samples. Prior to modeling, ASVs were grouped at phylum, family, and genus ranks and rare taxa (present in less than 1% of samples) were removed. Sample ASV counts were normalized using a centered log ratio transformation, with a pseudocount of 1 applied to all ASVs. Randomly generated training sample sets were made of 70% of each DD negative and positive samples, and testing sample sets contained the remaining 30% of samples in each group. Random forest models containing 1000 trees were trained using 100 repeats of 10-fold cross validation, and the number of variables sampled at each node was optimized as part of the caret R package v.6.0.86 (Kuhn, 2008). Model performance was evaluated based on accuracy of classification and kappa score, which was determined by constructing a confusion matrix based on the testing sample set. Relative variable importance along with relative abundance of each taxa was used to identify the microbiota that differentiate DD positive from DD negative samples. A *P* value of less than 0.05 was considered statistically significant for all comparisons. All analysis and figures were completed in R version 3.5.3.

## Results

### Study Selection and Characteristics

A total of 24 BioProject IDs were identified upon the initial search. Next, 12 studies were removed because they did not conduct deep amplicon sequencing of a 16S rRNA hypervariable region. Four studies were excluded because they were not from bovine skin/lesion samples, or did not have any raw fastq files associated with the BioProject ID. One study was removed because it did not use universal bacterial primers for sequencing. Finally, one study was removed due to a lack of metadata to differentiate samples by DD status. Included in the meta-analysis were 6 studies (**Table 1**), which all provided sufficient metadata to differentiate the DD status (DD negative samples, n = 37; DD positive samples, n = 190). The total number of raw sequences available for meta-analysis was 16,659,674, and after quality filtering, denoising, and merging, 8,827,942 reads were taxonomically classified. These samples were further categorized by sequencing platform, operation type (dairy, beef, or mixed, if known), 16S rRNA hypervariable region, DNA extraction kit, country of sample origin, and if the sample was known to come from an animal treated for DD. Samples that came from treated lesions (n = 16) were removed from the meta-analysis in order to focus solely on the differences in microbial populations between DD positive and DD negative skin.

### Characterization of Microbiota in DD Positive and DD Negative Skin

**Figure 1:**
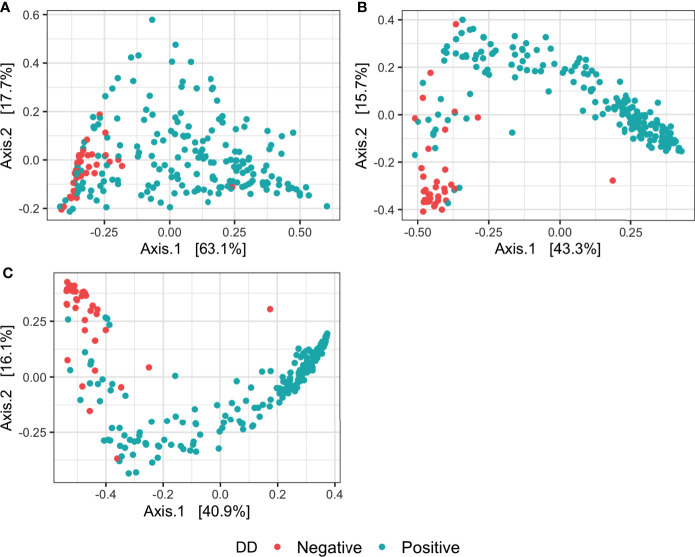
Principal coordinates analysis of Bray-Curtis distances of the microbiota in DD negative and DD positive skin. Bray-Curtis distances were calculated on samples with ASVs grouped at three different taxonomic levels: **(A)** Phylum, **(B)** Family, **(C)** Genus.

For all measures of alpha diversity tested, DD positive skin microbiota had significantly lower *(P* < 0.05) diversity, richness, and evenness when compared to DD negative skin microbiota (**Table 2**). Bray-Curtis distances between samples showed a significant difference *(P* < 0.05) in microbial composition between DD negative and DD positive skin (**Figure 1** and **Table 3**), regardless of the taxonomic rank tested. However, BETADISP was significant *(P* < 0.05) at the phylum level (**Table 3**). For these Bray-Curtis distances, the percent of variation explained by DD status increased as taxonomic rank lowered from phylum to genus (**Table 3**). When ASVs were grouped at the genus level, approximately 20.5% of the variation in microbial composition could be explained by DD status (**Table 3**). DD positive samples cluster in two major groups based on Bray-Curtis dissimilarities, and are visually separated by high and low *Spirochaetaceae* relative abundance (**Figure S1**).

**Table 2:** Alpha diversity estimates^a^ of DD negative and DD positive skin microbiota.

	Observed ASVs	Chao1	Shannon	Fisher
DD negative	384.5 ± 167.7	566.5 ± 354.0	5.2 ± 0.7	189.0 ± 137.6
DD positive	197.4 ± 144.5	244.6 ± 197.4	4.1± 1.1	74.6 ± 85.5
*P* value	4.3x10^-11^	3.8x10^-13^	1.9x10^-8^	3.3.x10^-10^

a Alpha diversity estimates shown are mean plus/minus standard deviation.

**Table 3:** Permutation analysis of variance and analysis of multivariate homogeneity of group dispersions on Bray-Curtis distances of DD negative and DD positive skin microbiota grouped at different taxonomic levels.

	PERMANOVA^a^			BETADISPER^b^	
	Pseudo-*F* ratio	R^2^	*P* value	*F* value	*P* value
Phylum	48.534	0.17874	0.001	12.384	0.001
Family	54.471	0.19631	0.001	0.1454	0.712
Genus	57.519	0.20504	0.001	2.653	0.114

a Permutational analysis of variance.

b Analysis of multivariate homogeneity of group dispersions.

**Figure 2:**
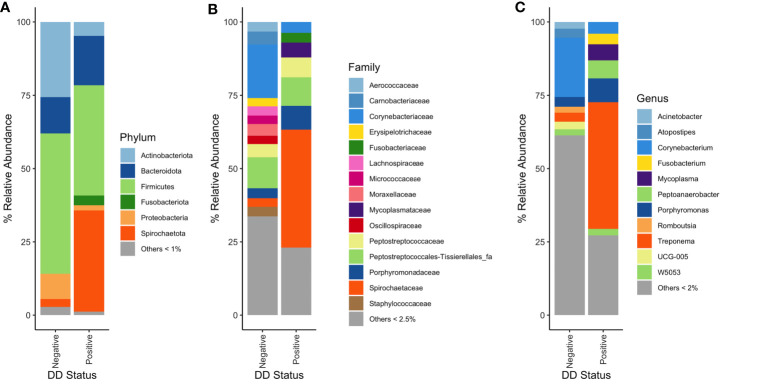
Mean percent relative abundances of microbiota in DD negative and DD positive skin. **(A)** Taxa were grouped at the phylum level, and all taxa that had less than 1% relative abundance were grouped. Taxa were grouped at the **(B)** family and **(C)** genus level, and all taxa that had less than 2.5% and 2% relative abundance, respectively, were grouped.

Distinct bacterial populations were found when DD negative skin microbiota was compared to DD positive skin microbiota. At the phylum level, *Spirochaetota* relative abundance was noticeably higher in DD positive skin, which was present at roughly 3% in DD negative skin to 35% relative abundance in DD positive skin (**Figure 2A**). There were also higher relative abundances of *Fusobacteriota*, *Bacteroidota*, and *Campilobacterota* in DD positive skin (**Figure 2A**). DD negative skin was dominated primarily by members of *Firmicutes*, *Actinobacteriota*, and *Proteobacteria* (**Figure 2A**). Additional trends emerged when increasing the taxonomic resolution to family and genus levels, as shown in **Figures 2B, C**. The families *Spirochaetaceae* and *Fusobacteriaceae* were higher in relative abundance in DD positive skin, similarly to their phylum level equivalents. In addition, families *Mycoplasmataceae* and *Porphyromonadaceae* were higher in relative abundance in DD positive skin compared to DD negative skin. At genus level grouping, *Treponema* dominated the DD positive skin microbiota; however, there were 4 other groups that also showed considerably higher relative abundance in DD positive skin compared to DD negative skin. *Fusobacterium* and *Peptoanaerobacter* were both relatively absent in DD negative skin, but were present at roughly 3% and 5% relative abundance in DD positive skin, respectively. *Porphyromonas* and *Mycoplasma* were present in DD negative skin at roughly 3% and 1%, respectively, but both had higher relative abundance in DD positive skin at greater than 6% relative abundance. In terms of bacterial prevalence, *Treponema, Porphyromonas, Mycoplasma, Fusobacterium*, and *Peptoanaerobacter* were present in the majority of DD positive skin samples, and were more prevalent in DD positive samples relative to DD negative skin samples (**Table S1**). *Mycoplasma, Treponema*, and *Porphyromonas* were all present in at least 94% of DD positive samples; whereas, *Peptoanaerobacter* and *Fusobacterium* were present in 81% and 63% of DD positive samples, respectively (**Table S1**). However, *Treponema* were also present in approximately 86% of DD negative samples, along with *Porphyromonas* and *Peptoanaerobacter* also present in more than 60% of DD negative samples (**Table S1**). *Amnipila*, *Ezakiella*, and *Campylobacter*, are genera that had low relative abundances at less than 1% in DD positive skin (**Figure 2C**), but were all present in greater than 75% of DD positive samples, while absent in the majority of DD negative skin samples (**Table S1**).

**Figure 3:**
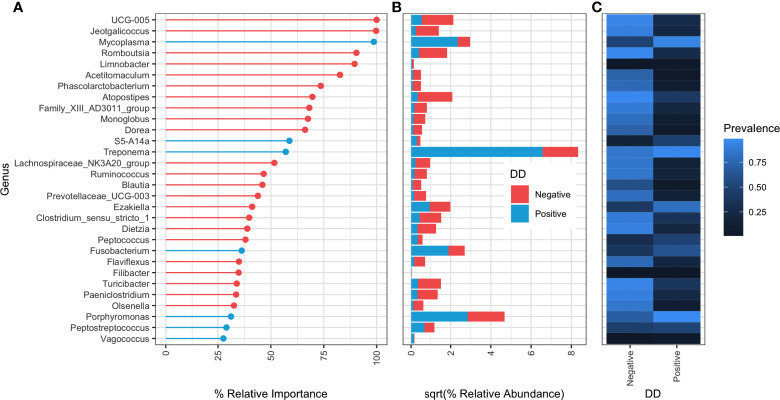
Relative importance ranking of random forest classifier at genus level taxonomy. **(A)** Percent relative importance of top 30 genera to the random forest classifier. Genera are coloured red if they have higher relative abundance in DD negative skin, and blue if they have a higher relative abundance in DD positive skin. **(B)** Square-root transformed relative abundances in DD positive and negative skin. Relative abundances were square-root transformed for easier visualization of large differences in relative abundance between genera. **(C)** Proportion of samples containing at least one sequence count of each genera.

Random forests classifiers built at each taxonomic rank all had at least 90% accuracy in classifying DD status based on microbial composition (**Table 4**). When bacterial taxonomy was grouped at the genus level, models had the highest accuracy at 97.06% and a kappa score of 0.82 (**Table 4**). Of the top 30 important genera in the random forest model, 23 were associated with DD negative skin (**Figures 3A–C**). *Mycoplasma* was the third ranked genus in the model, and was the first ranked DD positive-associated bacteria in the model. Six additional genera were designated as important markers of DD positive skin microbiota, of these, only *Treponema, Fusobacterium*, and *Porphyromonas* had relative abundances of greater than 3% in DD lesions, whereas the remaining 3 genera had a relative abundance of roughly 0.1% or less in DD positive skin.

**Table 4:** Evaluation of random forest model performance on classifying DD status from microbial composition at three taxonomic levels.

	Phylum	Family	Genus
Accuracy	0.90	0.91	0.97
Kappa	0.61	0.52	0.82

### Study Variables Influencing the Microbiota of DD Positive Skin

**Figure 4:**
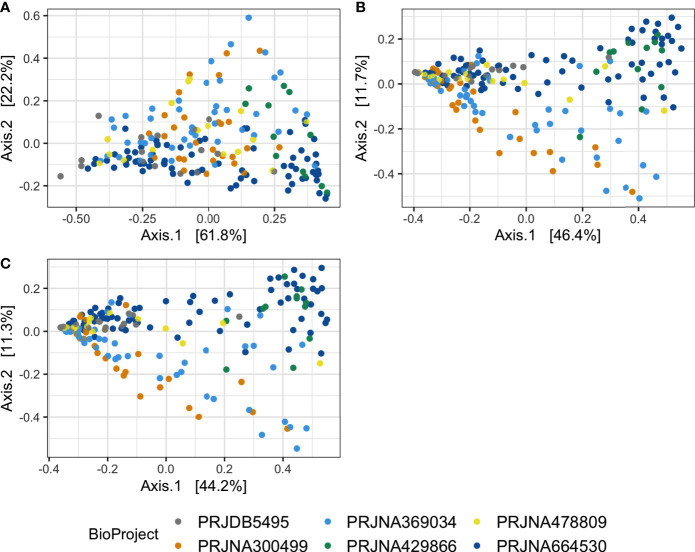
Principal coordinates analysis on Bray-Curtis distances of DD positive skin microbiota. Bray-Curtis distances were calculated on DD positive skin samples in which ASVs were grouped at **(A)** phylum, **(B)** family, and **(C)** genus level classifications.

Permutational analysis of variance on Bray-Curtis distances identified BioProject (individual studies) as the largest source of variation (20%) in microbial composition between DD positive samples, regardless of taxonomic levels tested (**Figure 4** and **Table 5**). The average relative abundances of non-rare taxa were relatively similar across studies, although one study detected distinctly lower relative abundance of *Spirochaetaceae* in DD positive samples (**Figure S2**). There were 3 different 16S rRNA hypervariable regions sequenced, and was another significant variable influencing the microbiota, accounting for roughly 11% of the differences between samples (**Table 5**). Sequencing platform, either Illumina or Life Sciences 454 pyrosequencing explained the lowest amount of variation of all categories tested (**Table 5**). All study variables, except for BioProject at phylum level grouping, displayed significant heterogenous dispersion across groups *(P* < 0.05) as identified in **Table 6**.

**Table 5:** Study variable PERMANOVA analysis on Bray-Curtis distances of DD positive skin microbiota grouped at different taxonomic levels.

Study variable	Phylum			Family			Genus		
	Pseudo-F ratio	R^2^	P value	Pseudo-F ratio	R^2^	P value	Pseudo-F ratio	R^2^	P value
BioProject (n=6)	9.5837	0.208	0.001	9.395	0.204	0.001	9.504	0.206	0.001
Amplicon (n=3)	12.466	0.118	0.001	11.852	0.113	0.001	12.23	0.116	0.001
Platform (n=2)	6.776	0.035	0.003	4.769	0.025	0.002	5.201	0.027	0.005

**Table 6:** Study variable BETADISP analysis of Bray-Curtis distances between DD positive skin microbiota grouped at different taxonomic levels.

Study variable	Phylum		Family		Genus	
	F value	P value	F value	P value	F value	P value
BioProject	1.969	0.086	5.225	0.002	4.829	0.001
Country	2.615	0.036	5.485	0.001	4.754	0.004
Amplicon	7.973	0.001	10.665	0.001	9.758	0.001
Platform	6.219	0.011	11.791	0.003	9.945	0.002

**Figure 5:**
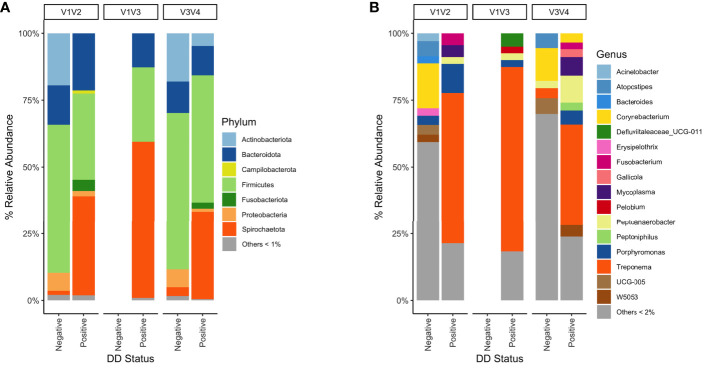
Mean percent relative abundance of DD negative skin and DD lesions from 16S rRNA hypervariable regions V1V2, V1V3, and V3V4. **(A)** Taxa were grouped at the phylum level, and all taxa that had less than 1% relative abundance were grouped. Taxa were grouped at the **(B)** genus level, and all taxa that had less than 2% relative abundance were grouped.

There were significant differences *(P* < 0.05) in richness, evenness, and diversity within DD positive samples from the V3V4 region compared to both V1V2 and V1V3 for all diversity measures evaluated (**Table 7**). No significant differences in any alpha diversity measurement existed between samples from V1V2 and V1V3 hypervariable regions (**Table 7**). In DD lesions from V1V3 amplification, there was a notable absence of *Fusobacterium* compared to the other two hypervariable regions, which show a *Fusobacterium* relative abundance of 2-4% in DD positive skin (**Figures 5A, B**). Although the proportions varied across sequencing regions, DD positive skin appeared to be dominated by *Treponema*, regardless of 16S rRNA hypervariable region (**Figure 5B**). Other bacterial genera with a relative abundance higher than 2% that were common across all sequencing regions in DD positive skin were *Mycoplasma*, *Peptoanaerobacter*, and *Porphyromonas* (**Figure 5B**). Although no DD negative skin samples were acquired from the V1V3 region, microbiota of DD negative skin from V1V2 and V3V4 sequencing both contained a community dominated by members of the phyla *Firmicutes*, *Actinobacteriota*, *Bacteroidota*, and *Proteobacteria* (**Figure 5A**).

**Table 7:** Alpha diversity estimates* of DD positive skin microbiota across different 16S rRNA hypervariable regions**.

	Observed	Chao1	Shannon	Fisher
V1V2	135.2 ± 132.5^a^	163.0 ± 198.2^a^	3.3 ± 0.6^a^	32.6 ± 58.3^a^
V1V3	98.4 ± 51.2^a^	105.5 ± 56.5^a^	3.1 ± 0.7^a^	19.5 ± 12.0^a^
V3V4	368.6 ± 157.3^b^	399.0 ± 172.2^b^	5.2 ± 0.7^b^	109.3 ± 70.3^b^

^*^Alpha diversity estimates shown are mean plus/minus standard deviation.

^**^Different letters within a column indicate significant difference (p < 0.05).

## Discussion

In this meta-analysis, *Treponema, Mycoplasma, Porphyromonas*, and *Fusobacterium* were the genera that best differentiated DD positive skin from DD negative skin, indicating their possible role in DD pathogenesis. These genera were present in the majority of DD affected animals, and were relatively more abundant in DD positive skin microbiota compared to DD negative skin. Whereas DD-associated microbiota and conclusions on which bacteria may have a role in DD etiology is varied across literature, the findings of this study identified a relatively consistent bacterial consortium in DD affected skin, regardless of study variables and experimental design choices. We also identify a consistent DD-associated microbiota across dairy and beef cattle breeds, providing justification for extrapolating dairy DD knowledge to beef cattle DD.

Although it was not possible to identify any taxa in 100% of animals with DD, there are strong associations of *Treponema, Mycoplasma, Fusobacterium*, and *Porphyromonas* with DD positive skin. This core group of genera we identified agrees best with the outcome of two previous metagenomic studies (Nielsen et al., 2016; Caddey et al., submitted for publication), in which they identify the same microbiota that best differentiates DD lesions from healthy skin. We identified other bacterial groups that displayed strong associations with DD skin, mainly members of *Firmicutes*, but these were left out of the primary core group because of their relatively low rank in the random forest model and low relative abundance in DD positive skin. However, low abundant taxa can have the capability to modulate community structures as keystone taxa (Banerjee et al., 2018), and ignoring taxa because of their low abundance may result in an incomplete picture of DD pathogenesis. Although it is unknown if low abundance taxa play a role in DD microbial community structure formation, we know *Dichelobacter nodosus* drives pathogenesis of ovine foot rot and only has a relative abundance of less than 2% in diseased tissue (Maboni et al., 2017), so it is possible that low abundant taxa may contribute to DD pathogenesis.

*Treponema* has been the most common genus implied as an etiologic agent of DD. Consistent with this study, virtually every other metagenomic study of DD observes a higher relative abundance of *Treponema* in most DD lesions compared to normal skin (Krull et al., 2014; Zinicola et al., 2015b; Marcatili et al., 2016; Nielsen et al., 2016; Hesseling et al., 2019). Conversely, it is not uncommon to see some healthy skin samples to also have high proportions of at least one species of *Treponema* (Beninger et al., 2018; Caddey et al., submitted for publication). Currently, there is little knowledge of pathogenesis mechanisms of individual *Treponema* spp., but *T. pedis, T. medium*, and *T. phagedenis* are some of the most commonly identified across DD literature (Klitgaard et al., 2013; Beninger et al., 2018), and may have pathogenic potential as identified in murine infection models (Elliott et al., 2007; Arrazuria et al., 2020).

*Mycoplasma* are more recently implicated in DD etiology (Krull et al., 2014; Nielsen et al., 2016), and they have been identified in this meta-analysis as the best DD-associated genus at differentiating disease status. There is a lack of understanding of which *Mycoplasma* spp. are important to DD development, due to a lack of culture isolates or species-specific identification methods, thus we only have short 16S rRNA amplicon-based identifications which do not give a definitive species identification. The major focus in literature tends to be on *M. fermentans* in DD pathogenesis, whose presence has been determined by PCR (Nielsen et al., 2016) and by bacterial culture from DD (Wyss et al., 2005). No DD studies have identified an abundance or presence of *M. bovis*, a major pathogen of bovine respiratory disease (Griffin et al., 2010). The lack of type cultures of *Mycoplasma* spp. isolated from DD lesions impairs our ability to further understand their roles in DD pathogenesis.

There is previous literature showing *Fusobacterium* as differentiating DD lesions from healthy skin (Sullivan et al., 2015; Nielsen et al., 2016; Moreira et al., 2018), but is suggested to have a larger role in chronic lesions due to it being more prevalent in those lesions (Hesseling et al., 2019). We did not identify *Fusobacterium* in any of the samples from V1V3 amplification. This could suggest that *Fusobacterium* is not important for lesion formation, and perhaps impacts severity of lesions as a secondary invader. However, there could be potential confounding effects that may have caused *Fusobacterium* to be absent in V1V3 reads, such as that only active lesion stages were sequenced, or there could be potential primer mismatches with DD-specific strains resulting in poor amplification efficiency. Two different *Fusobacterium* spp. have been identified and are both present in the majority of DD lesions of beef cattle (Caddey et al., submitted for publication); therefore, interest should remain on *Fusobacterium* as a potential pathogenic agent of DD. *Porphyromonas* is also a possible contributor to DD pathogenesis that is detectable in all active lesions in one study (Caddey et al., submitted for publication), but along with *Fusobacterium*, its superficial location in the dermis has led some studies to limit their conclusions on its potential involvement (Moreira et al., 2018). However, in periodontal disease, which has similar higher level microbial community structure to DD, *Porphyromonas* plays a significant role in influencing the metabolic activity of *Treponema* species (Tan et al., 2014). Additionally, *Porphyromonas* and *Fusobacterium* species generate mixed-species biofilms as a mechanism to impair the bovine innate immune system (Lockhart et al., 2017). While it is not evident that the same microbial strategies are involved in DD pathogenesis, it provides significant motivation to identify interactions between DD-associated species to determine a framework of the synergistic mechanisms used as pathogenesis mechanisms.

From the key group of DD-associated bacteria we mentioned in this study, multiple species within *Treponema, Porphyromonas*, and *Mycoplasma* have been identified within DD microbiota (Zinicola et al., 2015b; Nielsen et al., 2016; Beninger et al., 2018). Due to the unreliability of species level classifications of the 16S rRNA hypervariable regions sequenced in this meta-analysis, comparisons at the species level were not performed (Johnson et al., 2019). Methods to reliably quantify species-level dynamics across DD lesions is essential to validating these metagenomic results, and account for differences in 16S rRNA copy number and primer specificity between individual taxa.

One of the more common species implicated in DD lesion formation, *D. nodosus*, the etiologic agent of ovine foot rot, were present in less than 30% of DD samples in this meta-analysis (Rasmussen et al., 2012; Krull et al., 2014; Moreira et al., 2018). Insufficient amplification of *D. nodosus* isolates through one pair of universal 16S rRNA primers has been shown (Calvo-Bado et al., 2011), and could be a potential reason for *D. nodosus* absence in the majority of samples in this study. *Dichelobacter nodosus* is primarily suggested to have a potential role in early establishment of DD lesions, and then appears relatively rare in later lesion stages (Rasmussen et al., 2012; Krull et al., 2014), which could explain our inability to identify *D. nodosus* as clinically relevant. Further investigations of *D. nodosus* populations is warranted for early DD lesions, in order to characterize its potential role in epithelial infiltration and facilitation of skin colonization. Some studies not included in this meta-analysis have identified bacteria that show strong associations to DD lesion microbiota, such as *Candidatus Aemobophilus asiaticus*, which was present in large abundance in two studies (Zinicola et al., 2015a; Zinicola et al., 2015b), but not detected in this meta-analysis. Similarly, *Guggenheimella bovis* has been implicated in a potential DD pathogenesis role (Schlafer et al., 2008) but also was not identifiable in this meta-analysis.

Machine learning classifiers can help shed light on the complexity of polymicrobial infections that have major individual variation. These models appraise individual bacterial population dynamics with respect to overall community structure instead of the traditional statistical approach which primarily study individual taxa in isolation (Topçuoğlu et al., 2020). Random forest models provide a relative variable importance ranking, and in this meta-analysis, *Mycoplasma* was the best DD-associated bacteria at differentiating DD status, instead of *Treponema*, which is historically the most often group implicated in DD etiology. The relative variable importance is not a function of biological relevance of each organism, but rather scores variables based on their reduction of randomness in model prediction, and therefore we cannot conclude a relative ranking among DD-associated bacteria in their importance to DD pathogenesis.

Each of the studies included in this meta-analysis had significant differences in the microbial compositions of their samples based on PERMANOVA analysis. However, this could be due to the fact there was significant heterogenous dispersion between groups, so we cannot conclude that there are significant differences in the microbial composition between studies and study variables. The skin microbial composition and diversity of DD lesions is quite variable across individuals (Moreira et al., 2018), which is one of the reasons why it has been difficult to pin down a causative agent. This individual animal variation may be causing the heterogenous dispersions among groups tested for study bias.

In our meta-analysis, there were 2 studies that contained two-thirds of the total samples. These large studies may have skewed our results, but that is unlikely, given that our principal coordinates analysis appeared to show more diversity within studies than between studies, and it’s likely that the massive variation in *Treponema* abundance between samples was the source of this variation within studies. Observing the bacterial population differences between the 16S rRNA hypervariable regions identified minimal differences in the core bacterial group identified as potential DD pathogens, furthering evidence suggesting that these two large studies did not skew our results. An additional concern in study bias includes the significantly greater diversity in samples from V3V4 16S rRNA hypervariable regions compared to both V1V2 and V1V3. There are at least two potential reasons for this: 1) the samples that used V3V4 sequencing were also the only samples to include skin samples from beef cattle breeds rather than just dairy breeds; 2) V3V4 primers may amplify a wider range of bovine DD skin microbiota. DD in beef cattle is a recent field of study, as DD is emerging in those populations (Orsel et al., 2018), and since dairy and beef animals have separate housing environments and genetics, there is potential for their skin microbiota to have significant differences.

This meta-analysis has identified, through a consistent analytical approach of skin microbiota across multiple studies, that *Treponema*, *Mycoplasma, Porphyromonas*, and *Fusobacterium* were key genera that differentiated DD lesions from normal skin. These genera are consistently associated with the majority of skin samples of DD lesions. Based on abundance data, *Mycoplasma* best differentiates DD lesions from normal skin and should be a priority focus in future research on DD pathogenesis. *Treponema* are the most abundant bacteria in DD, but have also been identified in low amounts in most DD negative skin samples. Further analysis on the individual species of *Treponema*, as well as species of the other potential DD pathogens, is warranted to further understand their roles in DD lesions. In addition, longitudinal analysis of bacterial population dynamics throughout DD lesion progression would aid in elucidating potential causative roles of the DD-associated bacteria identified in this meta-analysis. This study is an accumulation of current understanding of DD microbiology, and provides strong evidence to standardize future research to focus primarily on the potential DD pathogens identified in this study.

## Data Availability Statement

The original contributions presented in the study are included in the article/**Supplementary Material**. Further inquiries can be directed to the corresponding author.

## Author Contributions

BC and JB conceptualized and designed the study. BC collected sequencing data and performed all data analysis. BC wrote the manuscript and JB edited and reviewed the manuscript. All authors contributed to the article and approved the submitted version.

## Funding

Funding was supplied by Alberta Agriculture and Forestry (grant number 2018F129R), the Canadian Dairy Commission under the Workplace Development Initiative, and the Simpson Ranch Grant at the University of Calgary Faculty of Veterinary Medicine.

## Conflict of Interest

The authors declare that the research was conducted in the absence of any commercial or financial relationships that could be construed as a potential conflict of interest.
