# Supplementary material for: Meta-Analysis of Bovine Digital Dermatitis Microbiota Reveals Distinct Microbial Community Structures Associated With Lesions
Source: Front Cell Infect Microbiol. 2021 Jul 16;11:685861. doi: 10.3389/fcimb.2021.685861 (PMC8322762; doi:10.3389/fcimb.2021.685861)
Supplement: Supplementary file 1 [file DataSheet_1.docx]

**Supplemental Material**


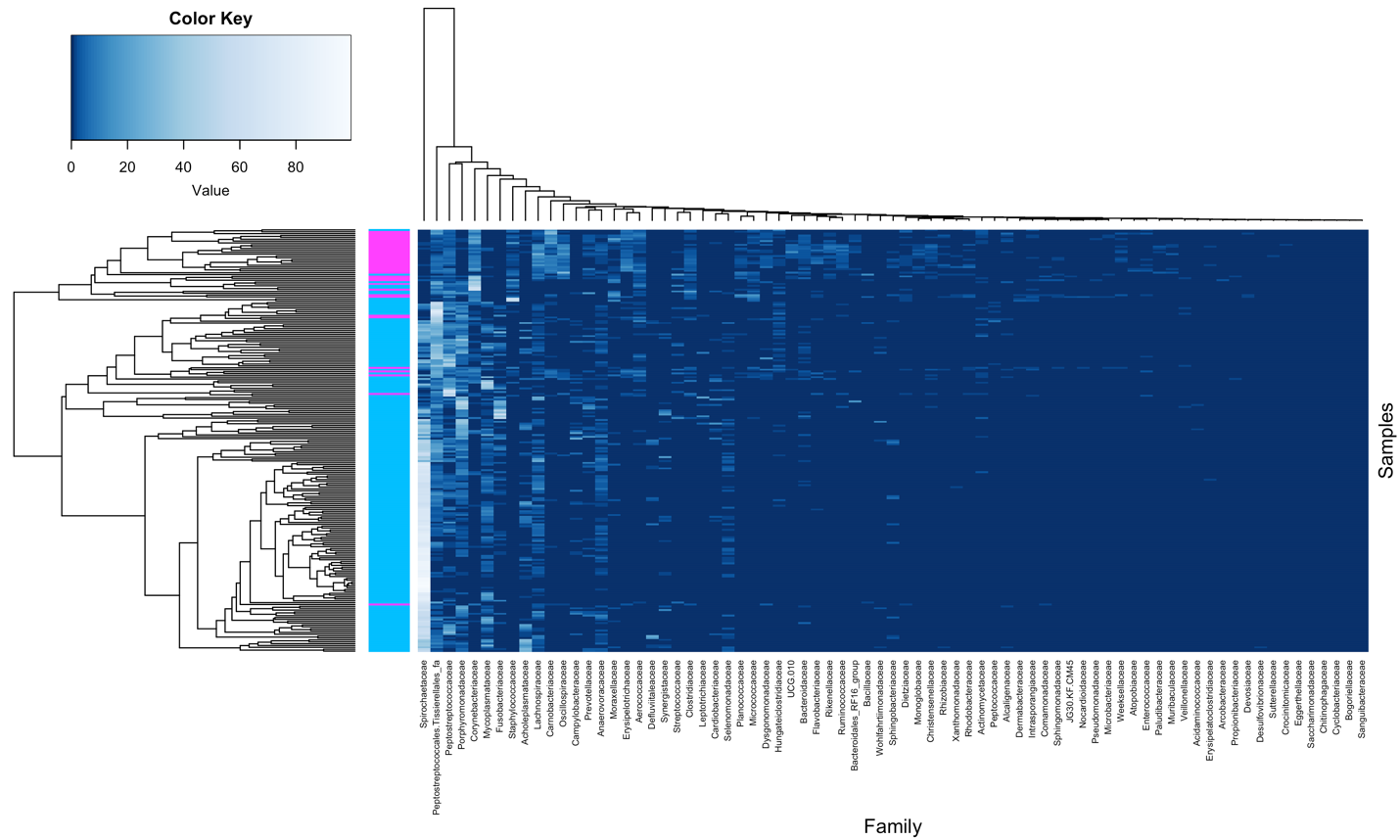


**Figure S1: Relative abundance heatmap of bacterial families on each sample included in the meta-analysis.** Rare families present in less than 10% of samples were removed from this analysis. Color key values represent percent relative abundance of each family within a sample. Dendrograms were built using hierarchical clustering on Bray-Curtis dissimilarities. Vertical color coding of samples represents DD status (Pink: DD negative; Light Blue: DD positive).


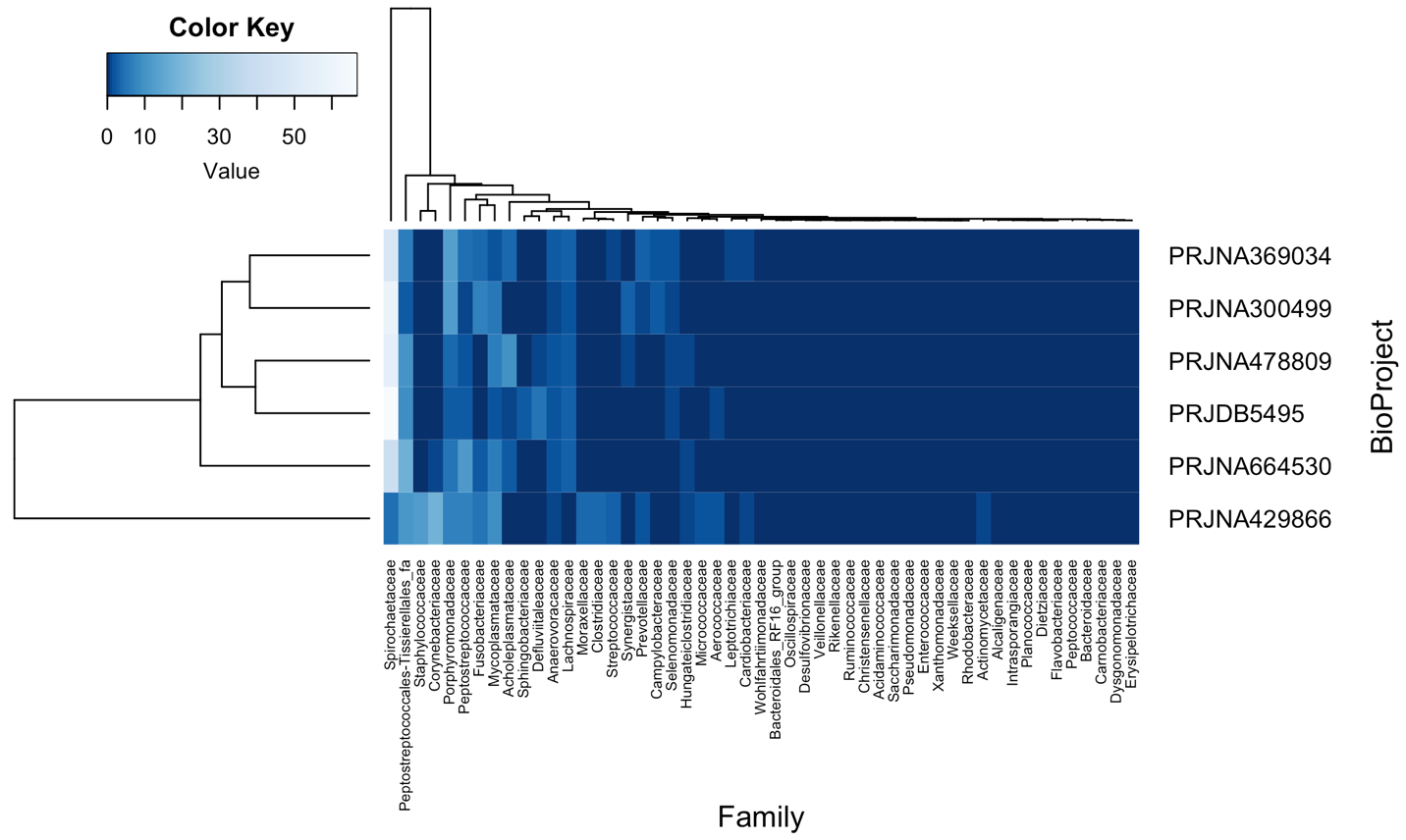


**Figure S2: Heatmap of average relative abundances of DD positive microbiota within each BioProject.** Only DD positive samples were included in the average relative abundance calculation within BioProjects. Taxa were grouped at family level, and rare families present in less than 10% of samples were removed from this analysis. Color key values represent average percent relative abundance of each family. Dendrograms were built using hierarchical clustering on Bray-Curtis dissimilarities.

**Table S1: Proportion of DD negative and DD positive skin samples containing at least one read of each genus**

| Genus | DD negative skin | DD positive  skin | Relative proportion in DD lesions^a^  (DD positive – DD negative) |
| --- | --- | --- | --- |
| Amnipila | 0.19 | 0.83 | 0.64 |
| Fretibacterium | 0 | 0.49 | 0.49 |
| Mycoplasma | 0.49 | 0.94 | 0.45 |
| Catonella | 0.11 | 0.56 | 0.45 |
| Ezakiella | 0.41 | 0.82 | 0.41 |
| Campylobacter | 0.43 | 0.75 | 0.32 |
| S5-A14a | 0.16 | 0.47 | 0.31 |
| Filifactor | 0.11 | 0.41 | 0.30 |
| Zymophilus | 0 | 0.30 | 0.30 |
| Porphyromonas | 0.70 | 0.98 | 0.28 |
| Fusobacterium | 0.38 | 0.63 | 0.25 |
| Negativicoccus | 0.05 | 0.3 | 0.25 |
| Centipeda | 0.08 | 0.31 | 0.23 |
| Anaerococcus | 0.51 | 0.73 | 0.22 |
| Parvimonas | 0.11 | 0.32 | 0.21 |
| Peptoanaerobacter | 0.62 | 0.81 | 0.19 |
| Peptococcus | 0.30 | 0.48 | 0.18 |
| Schwartzia | 0 | 0.15 | 0.15 |
| Gallicola | 0.62 | 0.75 | 0.13 |
| Peptoniphilus | 0.65 | 0.76 | 0.11 |
| Treponema | 0.86 | 0.96 | 0.10 |
| Desulfoplanes | 0 | 0.09 | 0.09 |
| Helcococcus | 0.65 | 0.73 | 0.08 |
| Wohlfahrtiimonas | 0 | 0.07 | 0.07 |
| Fenollaria | 0 | 0.07 | 0.07 |

^a^ top 25 genera ranked by difference in proportions of DD positive and DD negative skin samples are shown
